# Non-surface Attached Bacterial Aggregates: A Ubiquitous Third Lifestyle

**DOI:** 10.3389/fmicb.2020.557035

**Published:** 2020-12-04

**Authors:** Yu-Ming Cai

**Affiliations:** ^1^National Biofilms Innovation Centre, Institute for Life Sciences, University of Southampton, Southampton, United Kingdom; ^2^Biological Sciences, Institute for Life Sciences, University of Southampton, Southampton, United Kingdom

**Keywords:** bacterial aggregates, marine gel particles, marine/river/lake snow, activated sludges, chronic infection, *in vitro* models

## Abstract

Bacteria are now generally believed to adopt two main lifestyles: planktonic individuals, or surface-attached biofilms. However, in recent years medical microbiologists started to stress that suspended bacterial aggregates are a major form of bacterial communities in chronic infection sites. Despite sharing many similarities with surface-attached biofilms and are thus generally defined as biofilm-like aggregates, these non-attached clumps of cells *in vivo* show much smaller sizes and different formation mechanisms. Furthermore, *ex vivo* clinical isolates were frequently reported to be less attached to abiotic surfaces when compared to standard type strains. While this third lifestyle is starting to draw heavy attention in clinical studies, it has a long history in natural and environmental sciences. For example, marine gel particles formed by bacteria attachment to phytoplankton exopolymers have been well documented in oceans; large river and lake snows loaded with bacterial aggregates are frequently found in freshwater systems; multispecies bacterial “flocs” have long been used in wastewater treatment. This review focuses on non-attached aggregates found in a variety of natural and clinical settings, as well as some recent technical developments facilitating aggregate research. The aim is to summarise the characteristics of different types of bacterial aggregates, bridging the knowledge gap, provoking new perspectives for researchers from different fields, and highlighting the importance of more research input in this third lifestyle of bacteria closely relevant to our daily life.

## Introduction

The past 150 years have witnessed numerous breakthroughs in understanding the biology, biochemistry and ecology of free-swimming single celled planktonic bacteria following Louis Pasteur and Robert Koch’s seminal contributions. Clusters of bacterial cells on hard surfaces were observed by Leeuvanhoek as early as 1684, but the concept remained under explored for another 250 years. Then the 1930s embraced Paul Zobell’s extensive research on marine bacterial “films” on glass slides (Zobell and Allen, 1933; Zobell and Allen, 1935, Zobell and Anderson, 1936; Zobell, 1943), inspiring Bill Costerton to popularise the term “biofilm” to describe cells growing on surfaces as complex communities (Nickel et al., 1985). Ever since the concept that “the predominant lifestyle of bacteria found in natural settings are biofilms attached to surfaces” being brought forwards by the first generation biofilmers, research and progress has been mainly focussed on attached biofilms generated on different surfaces under specific conditions, such as the well-known mushroom structure in flow cell systems (Ma et al., 2009), or the crystal violet stained rings in microtiter plates (O’Toole, 2011). While these models are easily achievable *in vitro*, microbiologists from different fields started to question whether this mode is prevalent after examining samples from environment and patients. Recent studies raised the concept of a third lifestyle among medical microbiologists—non-attached bacterial clusters, which are proposed to be more prevalently found in chronic infections (Bjarnsholt et al., 2009, 2013; Secor et al., 2018). The term “aggregates” are applied to distinguish suspended clusters from surface-attached biofilms, which show some degree of similarity such as antibiotic tolerance, but also considerable differences in their phenotypes and regulatory mechanisms *in vivo*. As such, it is important to regard aggregates as a distinct existing format of bacteria more clinically relevant. However, suspended bacterial aggregates have been well studied in environmental science for many years. For instance, extensive studies on marine gel particles (MGP) and associated bacterial communities in sea surface waters, as well as the formation and fate of these aggregates during their sedimentation to the deep ocean were conducted by different groups (Zhou et al., 1998; Passow et al., 2001; Passow, 2002b; Engel, 2004; Cunliffe and Murrell, 2009; Cunliffe et al., 2009a,b; Ortega-Retuerta et al., 2009a; Galgani and Engel, 2013; Taylor et al., 2014; Busch et al., 2017; Engel et al., 2017; Mari et al., 2017; Zäncker et al., 2019). Large size activated sludges composed of bacteria and protozoa have been optimised and applied to the treatment of municipal wastewater (Sheng et al., 2010). Recognition of the ubiquitous nature of the aggregate lifestyle is now raising a number of fundamental questions: How are these non-attached bacterial aggregates formed? What are their functions both in nature and our bodies? Are there any fundamental similarities shared among aggregates formed in very different environments? Can we learn something from nature to help fight chronic diseases? Just as the pioneering marine work on biofilms led to the later clinical discoveries, the aggregates in marine, freshwater, wastewater and medical settings are summarised here, which may bring in inspirations on new directions of mechanistic studies, as well as more effective treatment strategies.

## *Finding Nemo*—Searching for the Bacterial Aggregates in Marine Gel Particles

**FIGURE 1:**
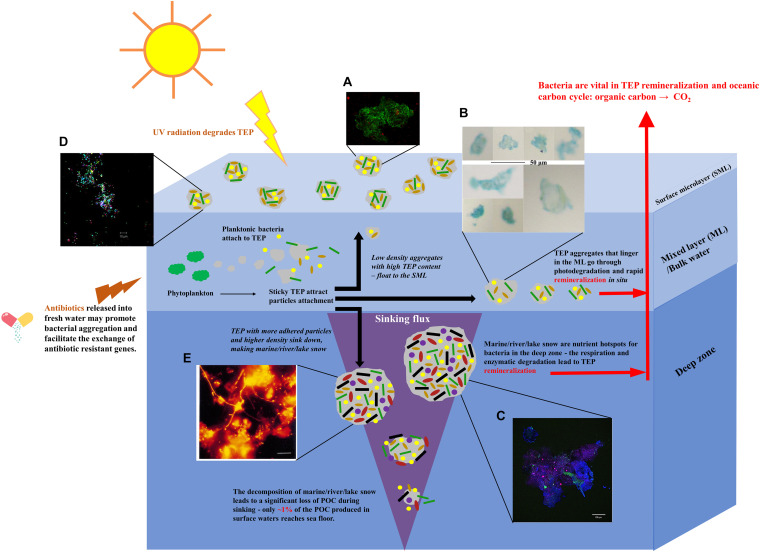
Schematic summary of the formation of bacterial aggregates in different aquatic systems, including oceans, rivers, lakes, ponds, and estuaries. Sticky TEP produced by phytoplankton provide accommodation for different bacteria, generating aggregates. Buoyant, lightweight aggregates with high TEP content float up to the SMLs. The majority of TEP stay in the ML and attract the attachment of bacteria/solid particles. They are quickly photolysed and remineralised. More adhesive TEP accommodate more particles that increase the overall density of aggregates. These aggregates sink down to the deep zones, forming marine/river/lake snow that become nutrient hotspots for bacteria in deeper zone (Grossart and Simon, 1998a,b). The fragmentation, enzymatic degradation, bacterial respiration, and remineralisation lead to the significant loss of TEP content. Image adapted from Cunliffe and Murrell (2009); Passow and Carlson (2012), and Herndl and Reinthaler (2013). Micrographs of **(A)** gel particle colonised with bacteria collected from sea SML (Engel et al., 2017). **(B)** Light microscopy micrograph of TEP obtained from bulk water (Zäncker et al., 2019). Scale bar = 50 μm. **(C)** An aggregate collected by the Marine Snow Catcher. Blue: polysaccharides; Green: nucleic acids; Red: chlorophyll (Busch et al., 2017). Scale bar = 100 μm. **(D)** Bacterial aggregates collected from SML in a freshwater pond (Cunliffe and Murrell, 2009). Scale bar = 10 μm. **(E)** Filamentous bacteria community of “river snow” stained with fluorescent *in situ* hybridisation (FISH) probes (Böckelmann et al., 2000). Scale bar = 10 μm. Written permissions to re-use images were obtained from respective authors, journals, and/or Copyright Clearance Centre when required.

Bacteria living in fluctuating aquatic environments, such as the ocean, do not necessarily live a single cell lifestyle like that experienced in shaken laboratory cultures. In fact, microorganisms in oceans readily inhabit MGP which provide physical surfaces, refuges, and chemical and nutrient gradients. These particles form aggregates at all depths from the ocean surface down to the bathypelagial region (Figure 1; Passow, 2002b; Busch et al., 2017; Zäncker et al., 2019). Two most common types of MGP found in the ocean are polysaccharide-containing Transparent Exopolymer Particles (TEP) and the proteinaceous Coomassie Stainable Particles (CSP). TEP are gel-like sticky particles predominantly composed of acidic polysaccharides, which are produced abiotically by the coagulation of dissolved or colloidal exopolymers released by phytoplankton (Passow, 2002b; Engel et al., 2004; Busch et al., 2017). In contrast, CSP is also gel-like, but less sticky than TEP, containing proteinaceous polymers released during cell death and decomposition (Long and Azam, 1996; Thornton, 2018). The sticky nature, as well as the abundance of carbon, nitrogen, and phosphorus components render TEP and CSP ideal habitants for microbial cells (Zäncker et al., 2017), which can use them either as pure attachment sites or nutrient resources (Zäncker et al., 2019). The precursor materials, such as polysaccharides produced by phytoplankton living in the ocean mixed layer, serve as a major substratum of TEP and CSP aggregation. Bacteria loaded TEP aggregates can either float to the sea surface microlayer (SML) due to their buoyant nature or sink down to the deeper zones when the size and density are increased with more attached microorganisms or other particles, forming marine snow that transports nutrients. While TEP has been extensively studied due to its ubiquity and the importance in marine carbon cycle (Mari et al., 2017), research on CSP is still at the embryonic stage (Busch et al., 2017). Here, bacterial communities co-aggregated with these two types of marine particle are discussed.

### Aggregates in the Sea Surface Microlayer (SML)

SML is considered to be roughly the upper most 1 mm of the ocean at the air–sea interface (Cunliffe and Murrell, 2009). Acting as the ocean’s skin, it controls the exchange of gas, energy and matter between the air and the sea. It contains a variety of organic and inorganic matter providing accommodation for bacteria communities referred to as bacterioneuston (Figure 1A; Engel et al., 2017). The growing studies of bacterioneuston and the acknowledgment of the accumulation of gel particles has led to the concept that the SML can in many ways be regarded as a gelatinous biofilm (first proposed by John McN. Sieburth in 1983) (Sieburth, 1983; Wurl and Holmes, 2008; Cunliffe and Murrell, 2009; Flemming and Wuertz, 2019). This layer is enriched by TEP and CSP particles as has been observed in different ocean systems (Wurl et al., 2011; Engel and Galgani, 2016; Zäncker et al., 2017). Although the resource of dissolved polysaccharides mainly comes from the phytoplankton in the upper water column, especially after blooms, the low density and the positive buoyancy can raise TEP up to the SML. The SML may also be enriched with high-molecular organic compounds on the surfaces of bubbles, which are potentially more surface-active and tend to polymerise (Johnson and Cooke, 1980; Gershey, 1983; Flemming et al., 2016). This proposal was supported by the fact that TEP was generated by bubbles that were rich in covalently bound sulfate (Zhou et al., 1998), which is consistent with the observation that TEP in the SML contains a higher fraction of half-ester sulphate bonding molecules than in the sub-surface water (Wurl and Holmes, 2008).

The enrichment of TEP may explain the existence of large numbers of bacteria in the SML. Apart from the gel-bacteria coagulation effect and providing nutrients, TEP may also shelter cells from the detrimental effects of factors like UV radiation, in a similar way to that of extracellular polymeric substances (EPS) which protect microbial cells in biofilms (Yin et al., 2019). Ortega-Retuerta et al. (2009a) experimentally determined how ultraviolet B (UVB) radiation influenced the degradation of TEP using both natural sea water and batch culture of diatom species producing large amount of TEP. A significant TEP photolysis was observed, with loss rates from 27 to 34% per day. Interestingly, when natural sea water microorganisms were present, TEP increased greatly under UVB radiation, suggesting that UV promotes the production of TEP by microorganisms (Ortega-Retuerta et al., 2009a). As such, bacterioneuston and TEP may mutually protect each other from the strong UV radiation occurring in the SML, and the release of TEP in the bacteria rich SML needs further attention for its role in carbon exchange.

The diversity and abundance of the bacterioneuston have been studied in recent years (Cunliffe et al., 2011; Engel et al., 2017). Different species have been discovered in SML from different locations (Franklin et al., 2005; Obernosterer et al., 2008; Cunliffe et al., 2009b; Stolle et al., 2011; Taylor et al., 2014). However, despite the widely accepted importance of TEP as the matrix holding the bacterioneuston together, the diversity of bacterial communities that specifically or preferably attach to TEP have not been widely investigated (Zäncker et al., 2019). For instance, Taylor et al. (2014) observed that an increased abundance of *Flavobacteriales* and *Rhodobacterales* belonging to *Bacteroidetes* and *Alpha-proteobacteria* at English Channel time-series station L4 was significantly related to the decline of TEP concentration, suggesting a role of both taxa in TEP utilisation. Stolle et al. (2011) specifically compared both the non-attached and particle-attached bacterial assemblages in the Southern Baltic SML using 16S rRNA. Different strains belonging to *Bacteroidetes, Cyanobacteria, Alpha-, Beta-*, and *Gammaproteobacteria* were all found attached to particles. Obernosterer et al. (2008) also observed *Bacteroidetes* and *Gammaproteobacteria* dominating SML in the South Pacific Ocean. However, larger sample sizes collected from different locations are needed to better summarise the most prevalent orders and families associated with TEP in SML.

While many studies focussed on TEP enrichment in SML, experiments from Galgani and Engel using seawater samples from the North Sea and the diatom *Thalassiosira weissflogii* demonstrated an enrichment of CSP that dominated the gelatinous SML (Galgani and Engel, 2013). While bacterial abundance in SML may be strongly influenced by interactions with CSP, little is known about the diversity of bacteria attached to CSP. More knowledge of the ecology of the bacterial species specifically attaching to different particles from various locations are required for a more comprehensive picture. The results may be largely dependent on the sampling method and which parts of the SML are reached as some techniques may be biased towards specific cell types (Cunliffe and Murrell, 2009). Hence, a full comparison of all SML sampling methods would allow a better understanding of the limitations of each method and a better interpretation of the results.

### Bacterial Colonisation on Particles in the Mixed Layer

The ocean mixed layer (ML) is a region of seawater situated adjacent to the air–sea interface which is subject to continuous mixing, and has relatively uniform temperature and salinity (Kantha and Anne Clayson, 2004). The pre-cursor materials for TEP, such as polysaccharides, are mainly produced by phytoplankton in the ML which is directly related to the concentration of TEP in this same layer (Ortega-Retuerta et al., 2009b; Wurl et al., 2011; Van Oostende et al., 2012). A large discrepancy was observed between the removal of dissolved inorganic carbon in the surface water and downward flux of particulate organic carbon, and the formation of carbon rich TEP generating a C-rich pool was proposed to explain this phenomenon (Sambrotto et al., 1993; Michaels et al., 1994; Schartau et al., 2007). The ML locates between the SML and the deep sea, and TEP newly produced in this area face three options for relocation—moving up to the SML, staying in the ML, and sinking down to the deeper zone. How TEP move therefore plays an important role in the oceanic carbon cycle. The density of TEP is lower than that of seawater, but due to its sticky nature the overall density of TEP aggregates can be greatly changed with the solid particles glued to them (Engel and Schartau, 1999; Azetsu-Scott and Passow, 2004). The fraction of carbon content contributed by TEP to the whole aggregate determines the degree of retention and remineralisation in surface waters vs. its downward export (Mari et al., 2017). It is now hypothesised that most TEP aggregates readily linger in surface waters, and they only sink when ballasted with high-density particles (Mari et al., 2017). In addition, the accumulation of TEP in the ML also depends on the balance between production and removal. While the main source of TEP is phytoplankton, the frequently produced EPS from bacteria can also contribute to the formation of TEP (Stoderegger and Herndl, 1999; Passow, 2002a; Sugimoto et al., 2007; Wurl et al., 2011). On the other hand, the extracellular enzymes released by bacteria attached to TEP aggregates, such as β-glucosidase and LAPase, result in the degradation and remineralisation of TEP (Arnosti et al., 2005, 2009; Bar-Zeev and Rahav, 2015; Burns et al., 2019). The abundance and species of bacteria communities colonised on the particle surfaces heavily influence the degradation of TEP aggregates in the ML. Consequently, the microscale community ecology of bacteria on particle surfaces plays an important role in the rates of carbon turnover in the ocean (Enke et al., 2018).

Despite many reports on the observation of TEP in ML from different oceans (Flemming et al., 2016), current knowledge mainly points to the abundance of bacteria associated with TEP rather than the diversity. For example, Mari and Kiørboe (1996) reported that bacterial abundance was positively related to the volume concentration of TEP sampled from the ML of Kattegat. Busch et al. (2017) analysed bacterial colonisation on both TEP and CSP in different ocean layers of the Arctic Fram Strait. The highest concentrations of bacteria attached to gel particles were observed in surface waters (≤100 m) at all stations and on both types of gel particle. TEP had the highest bacterial concentrations within the upper 30 m in most locations. However, neither study revealed the taxonomy inhabiting the particles. A recent study from Zäncker identified two operational taxonomic units, *Marinobacter adhaerens* and *Glaciecola sp.* Within the *Alteromonadaceae* family, that abundantly attached to TEP in the total bulk water sampled from Plymouth Sound (United Kingdom) (Zäncker et al., 2019; Figure 1B). Note that *Alteromonadaceae* does not belong to the frequently found families from SML at the other locations mentioned above. Whether the bacterial communities change drastically during ascending to SML is waiting for more detailed exploration by comparing the taxonomies presented both from SML and ML at same locations.

The fact that CSP are abundant in cultures of diatoms, cyanobacteria, and heterotrophic bacteria suggest that these organisms may also serve as a significant source of CSP in the ML (Thornton, 2018). However, little is known about whether organisms from these phyla are attached to the CSP sampled from the ocean. Heterotrophic bacteria attached to CSP may also be utilising the proteinaceous nutrients, and may account for the decreasing vertical depth profile in CSP abundance that was observed (Busch et al., 2017). Hence, the interactions between the bacteria and CSP concentrations remain to be elucidated but are likely to be complex (Thornton, 2018).

Although ML is probably where the majority of TEP and CSP originate, the diversity of bacterial communities attached to these MGPs, as well as the interaction and influence between them in a broader spectrum of locations, remain unknown. Further study using molecular techniques to identify the taxonomic patterns that occur within the MGPs is required in the future.

### Bacterial Load on Marine Snow

The majority of TEP are of low density and retained in the euphotic zone followed by UV radiation-mediated photodegradation and *in situ* remineralisation (Mari et al., 2017). However, some adhesive TEP can adsorb organic matter and mineral elements, capture living organisms and promote aggregation, resulting in an increase in size and density and the production of large aggregates that sink down to the deeper zone, known as “marine snow” (Passow, 2002b; Bittar et al., 2018). Due to their high organic matter content, marine snow aggregates become nutrient hotspots for particle-attached bacteria (Flintrop et al., 2018). The microbial degradation of organic matters greatly influence the vertical transfer of carbon sources from the surface to the mesopelagic and deep ocean during the sinking of marine snow (Passow et al., 2001; Bittar et al., 2018; Flintrop et al., 2018). Furthermore, the consequence of this hydrolysis may result in a leak of material into surrounding water and generate nutrient gradients around the particles, extending the benefits to the free-floating microbes nearby and potentially increasing bacterial production (Stocker and Seymour, 2012). Consumption by zooplankton and microorganisms, bacterial respiration and enzyme-mediated remineralisation, as well as fragmentation altogether result in the fact that the portion of particulate organic carbon transported into mesopelagic zone decreases with depth, which can reduce the export flux by ∼70–90% (Passow and Carlson, 2012; Collins et al., 2015; Briggs et al., 2020). In the end, only ∼1% of the particulate organic carbon produced in surface water reaches the sea floor, and the remineralisation throughout the sinking converts the organic carbon back to CO_2_ (Herndl and Reinthaler, 2013). As such, the taxa and activities of bacteria associated with marine snow aggregates in the deep zone are again vital for the global carbon cycle.

Several studies have addressed the abundance of bacterial communities inhabiting marine snow (Figure 1C). Marine snow collected from different locations showed a great variance in microbial abundance (Simon et al., 1990; Thiele et al., 2015). It was reported that microbial abundance on marine snow aggregates in the northern Adriatic Sea was much higher (10^3^–10^4^ times) than the concentration of planktonic cells in the surrounding seawater (Ivancic et al., 2018). This difference in microbial abundance between marine snow aggregates and free-living bacteria in bulk water was also observed at Cape Blanc at both 100 and 400 m depth (Thiele et al., 2015), indicating a potential general trend in all oceans.

Marine snow collected from surface waters were reported to be dominated by *Alpha*-, *Gamma*-*Proteobacteria* and organisms belonging to the *Cytophaga-Flavobacterium-Bacteroides* complex (DeLong et al., 1993; Gram et al., 2002). Some gram positive bacteria such as *Bacillus* and *Clostridium* were also isolated (Gram et al., 2002). However, the microbial composition in surface water marine snow may not be similar to the ones in deeper zones. Due to the difficulty in sampling, so far little is known about bacterial diversity associated with sinking marine snow. A recent study from Thiele et al. (2015) specified that the bacteria colonised on marine snow in the twilight zone belong to *Roseobacter, Bacteroidetes, Alteromonas, Pseudoalteromonas, Planctomycetes, Synechococcus*. There may be differences in marine snow bacterial composition between surface waters and deep ocean, but more samples are needed for wider comparisons.

As mentioned above, two of the limitations for the investigation of bacterial communities in oceans are achieving intact sampling and the deployment of *in situ* observation techniques. While the use of Marine Snow Catcher water sampling devices, or drifting sediment traps equipped with viscous cryogel embedding media are useful for the collection of intact marine snow (Thiele et al., 2015; Busch et al., 2017), a new method was recently developed by Flintrop et al. (2018). This uses a soft-embedding technique for the rapid quantitation of bacteria followed by sectioning with fluorescence *in situ* hybridisation (FISH) to study the spatial structure and components of the marine aggregates at high resolution (Flintrop et al., 2018). Future studies may focus on investigating marine snow obtained from different oceans with the combination of these techniques.

## *Some Like It Fresh*—Aggregated Bacterial Communities in Freshwater Systems

### Lakes and Ponds

One of the most extreme examples of how microorganisms can degrade freshwater quality for recreation and drinking water, as well as threatening fisheries and human health is the phenomena of cyanobacterial blooms (Huisman et al., 2018). Cyanobacterial surface blooms are often dominated by cyanobacterial aggregates (CA) formed by buoyant genera such as *Aphanizomenon, Cylindrospermopsis, Dolichospermum, Microcystis, Nodularia*, and *Planktothrix* (Huisman et al., 2018). CA are normally held together by extracellular polysaccharides released from themselves to form a phycosphere, similar to surface-attached biofilms (Zhu et al., 2019). However, cyanobacteria are not the only occupants of these large CAs. Just as TEP are mainly generated by phytoplankton but capture different species of bacteria, CAs are also extensively colonised by heterotrophic bacteria providing key nutrients (Cai et al., 2014; Woodhouse et al., 2018; Zhu et al., 2019). At least 22 bacterial divisions were found associated with CAs sampled in Lake Taihu, China, with the dominating two phyla *Proteobacteria* and *Bacteroidetes* (Cai et al., 2014). A later study from Zhu et al. (2019) revealed that *Microcystis* and *Dolichospermum* alternately dominate CAs in the same lake. Specific bacterial attachment pairs were observed for *Dolichospermum-Burkholderia* and *Microcystis-Hyphomonas*, suggesting that the co-habiting bacteria found in CAs are more correlated to the cyanobacterial content than to the specific environments (Zhu et al., 2019). Samples from a shallow eutrophic pond showed that cyanobacterial associated microbial assemblages were mainly from stable dominant taxa. A large number of bacterial-bacterial correlations were observed between these stable taxa which may be due to a high inter-dependency (Woodhouse et al., 2018). Therefore, it may be concluded that the dominant bacteria species attached to CAs in a specific freshwater system are more dependent on the resource of CAs rather than the environmental changes.

Bacteria can colonise organic matter in lakes, generating aggregates similar to those of marine snow, but called “lake snow” (Grossart and Simon, 1998a). Despite the fairly small sizes, “lake snow” aggregates can be densely colonised by bacteria with the abundance 100 × higher than in the bulk water (Grossart and Simon, 1993). Weiss et al. (1996) found all α-, β-, and γ-subclasses of *Proteobacteria* on “lake snow” aggregates in Lake Constance (Germany), with *β-*subclass always dominating the bacterial communities. A particular type of iron-rich lakes aggregates observed in a stratified lignite mine lake in Germany, termed as “iron snow,” has been highlighted by Küsel group (Reiche et al., 2011). Iron snow associated bacteria so far isolated have been phylogenetically identified as acidophilic Fe(II)-oxidising bacteria related to *Acidithrix (Actinobacteria)* or *Ferrovum (β-Proteobacteria)*, as well as acidophilic heterotrophic Fe(III)-reducing bacteria related to *Acidiphilium, Acidisphaera*, and *Acidocella (α-Proteobacteria*) (Lu et al., 2013; Mori et al., 2017). These results indicated that the organic or inorganic composition of the aggregate matrix can be significantly influenced by the environmental conditions and the selective dominating species, which in turn influence the taxonomic patterns of the bacteria attached to them. However, what is the general component of “lake snow” matrix, as well as how it is formed and consumed needs further research at different locations.

Bacterial aggregates are also found in ponds. Kirchman analysed the seasonal change of bacterial communities in a pond and found that particle-bound bacteria were relatively more abundant from July to October than during the cold winter months, with less than 10% of the total population attached to particles at any time (Kirchman, 1983). Alfiansah et al. (2018) reported a dominant role for *Halomonas* and *Psychrobacter* genera in the particle-attached communities in a shrimp aquaculture pond. Much bacterial aggregation was observed in the SML water collected from a freshwater pond on the University of Warwick campus (Figure 1D; Cunliffe and Murrell, 2009), suggesting that bacterial aggregation in the ponds resembles, at least to some extent, the distribution of bacteria seen in the marine system.

### River and Estuary

Lotic microbial aggregates, termed “river snow,” have also been documented (Böckelmann et al., 2000; Neu, 2000). The bacterial communities of “river snow” in the Elbe River (Germany) were reported to change in different seasons, showing a great diversity in spring and summer but a reduction of the total cell count was recorded in autumn and winter (Böckelmann et al., 2000). In contrast to the particle-attached taxa in the Columbia Estuary, but similar to the “lake snow” in Lake Constance, β-*Proteobacteria* had a dominating role, forming up to 54% of the total cell counts. Other lineages such as α- and γ-*Proteobacteria*, *Planctomycetales*, and *Cytophaga-Flavobacteria* were also detected *in situ* in Elbe “river snow” (Figure 1E; Böckelmann et al., 2000).

Estuaries, where freshwater meets saltwater, also witness a large number of particle-attached bacteria, sometimes making up more than fifty percent of the total community. In the Columbia Estuary (United States), most particle-attached bacterial taxa were related to members of the genus *Cytophaga* or the α-, γ-, or δ- subclasses of *Proteobacteria*, while *b-proteobacteria*, gram-positive bacteria, and *Verrucomicrobium* spp. mainly retained a free-living style (Crump et al., 1999). Constantly higher TEP concentrations were observed at the bottom of the Qishon estuary (Israel), heavily packed with numerous heterotropic bacterial clusters (Bar-Zeev and Rahav, 2015). Campbell and Kirchman (2013) reported that the dominating bacterial taxa in the Delaware Bay (United States) samples can change greatly along the salinity gradient in estuary, from typical fresh water communities such as *Actinobacteria, Verrucomircobia*, and *Betaproteobacteria* to typical marine communities such as *Rhodobacterales, Gammaproteobacteria*, and *Bacteroidetes*, confirming that aquatic environment plays dominant role in bacterial composition.

Apart from the need for more sampling to be carried out at other locations to further reveal the scope of bacterial interactions within lake and river snow, another serious issue has been raised recently relating to bacterial aggregation in freshwater. It is well known that antibiotic resistant genes (ARG)/isolates have been widely observed in different freshwater systems (Ash et al., 2002; Kim et al., 2015; Yang et al., 2018; Zhu et al., 2017; Nnadozie and Odume, 2019), but now sulphonamide and tetracycline resistant genes have been shown to have significantly higher abundance in particle-attached bacteria than free-living ones in the Zenne River (Belgium) (Proia et al., 2018). Guo et al. (2018) reported that the antibiotic resistant community of free-living bacteria can be lowered during cyanobacterial blooms, but that in the particle-attached bacterial community they maintain the same abundance. This indicated that the bacteria aggregates associated with particles encouraged the stability of ARGs in the environment, which is related to the widely accepted concept that biofilms are hot spots for the horizontal transfer of ARGs, and the observations that cells encased in biofilms show much higher tolerant to antibiotics. To make the problems even more complicated, antibiotics were documented to promote the aggregation of freshwater bacterial strains in continuous laboratory cultures using artificial lake water medium (Corno et al., 2014). This phenomenon may reflect the real-life situation where the antibiotics released into freshwater facilitate the co-aggregation of resistant and non-resistant isolates, which in turn results in the exchange of ARGs among strains. Once the aggregates are degraded, new resistant isolates are dispersed and spread with the potential for gene modifications to also take place. Hence, investigations into the influence of antibiotics on bacterial aggregation in different freshwater systems are urgently required.

## *Sewage Millionaire*—Flocs in Wastewater Treatment

When our last night dinners are flushed down the toilet, they go on feeding the bacteria in sewage water. As in other water systems, bacteria can form multi-cellular aggregates (also known as flocs), which have been extensively studied in the activated sludges (AS) wastewater treatment plants (Jorand et al., 1995; Zita and Hermansson, 1997; Da Motta et al., 2001; Wilén et al., 2003; Nielsen et al., 2004; Larsen et al., 2008; Foladori et al., 2010). The AS process is the most prevalently applied biological wastewater treatment method, where suspended bacterial aggregates are used for removing pollutants based on their capacity to break down organic carbon substances and consume nutrients such as nitrogen and phosphorus (Gernaey and Sin, 2008).

**FIGURE 2:**
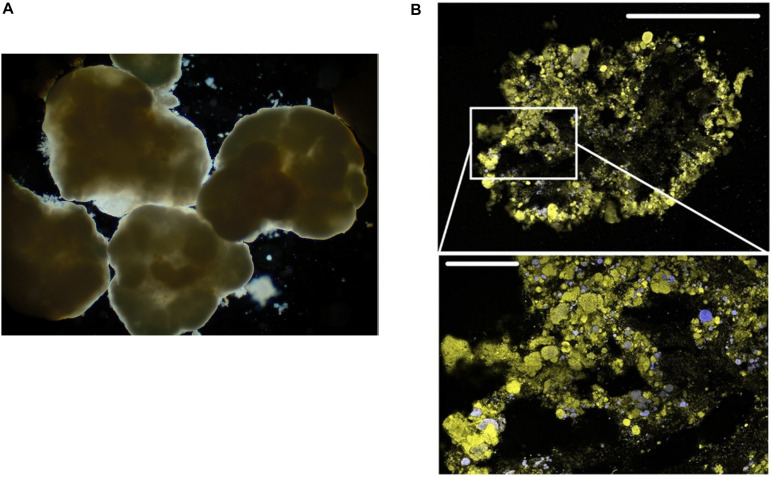
**(A)** Light microscopy micrograph of aerobic granular sludge (Wilén et al., 2018). **(B)** FISH-CLSM images from cryosections of aerobic granular sludge (upper scale bar: 500 μm) and detailed sections (lower scale bar: 100 μm). Yellow: total cells; blue: ammonia oxidising bacteria (Szabó et al., 2017). Written permissions to re-use images were obtained from journals.

Sludge flocs are mainly composed of microorganisms stuck together with EPS matrix, organic, and/or inorganic colloidal particles (Figure 2A; Urbain et al., 1993; Sanin and Vesilind, 1996; Bitton, 2010). Considerable attention has been paid to (1) the structure and the physico-chemical properties of flocs (Andreadakis, 1993; Snidaro et al., 1997; Wilen and Balmer, 1999; Sponza, 2002; Jin et al., 2003; Nielsen et al., 2004; Kara et al., 2008); (2) isolation and characterisation of floc forming bacteria (Nielsen et al., 2004), both important in sludge management such as flocculation, settling, dewaterability and effluent quality (Nielsen et al., 2004). Bacteria constitute 5–20% of organics in flocs, of which filamentous bacteria plays a significant role (Raszka et al., 2006; Nielsen et al., 2010). Both auto-aggregation (genetically identical cells forming flocs themselves) (Trunk et al., 2018) and co-aggregation (adherence of genetically different cells) (Malik et al., 2003) happen in flocs. The phylum *Proteobacteria* was documented to be dominant, with β-*Proteobacteria* being the most abundant class for their important roles in both producing flocs EPS (such as *Zoogloea*; Rossello-Mora et al., 1995; Shao et al., 2009) and in organic matter degradation (Nascimento et al., 2018). α-, γ-, δ- *Proteobacteria, Actinobacteria, Bacteroidetes, Firmicutes, Chloroflexi* were also found but less abundant (Nielsen et al., 2004, 2010; Larsen et al., 2008; He et al., 2016). More specifically, some core genera including *Zoogloea, Dechloromonas, Prosthecobacter, Caldilinea, Tricoccus, Gp4*, and *Gp6* in *Acidobacteria* and Subdivision3 genera *incertae sedis of Verrucomicrobia* have been identified from all 14 activated sludge in different locations (mainland China, Hong Kong, Singapore, Canada, and United States) (Zhang et al., 2012), indicating their importance in maintaining the normal function of wastewater treatment plants.

Knowing the composition and function of bacteria communities facilitates the development of more efficient wastewater treatment methods. While AS has served as a standard technology for the biological treatment of sewage, it does have some drawbacks. For instance, the poor settling ability of active-sludge can result in the excessive loss of sludge in the effluent, thus significantly lowering the effluent quality. Furthermore, uncontrolled sludge ages can gradually lead to the loss of treatment efficiency (Nancharaiah and Kiran Kumar Reddy, 2018). A relatively advanced strategy, aerobic granular sludge (AGS), has been applied for the efficient removal of organic carbon, ammonium and nitrogen etc. (Figure 2B; Szabó et al., 2017). AGS is generated by inoculating AS to the column reactors operated in sequencing batch reactor mode with bubble aeration and short settling times. The seeded AS are first induced to form large aggregates in the reactors. Then, by repeatedly operating sequencing batch reactor with short settling time, the sludges with poor settling ability are washed out, while aggregates with rapid settling time are selected and kept for further growth. Eventually, this selection and separation resulted in large-size granular sludges with high density allowing for rapid sediment, and high EPS content improving the stability and providing cells inside with higher tolerance to the toxic pollutants (Nancharaiah and Kiran Kumar Reddy, 2018).

Some core genera have been identified in different AGS, including but not limited to *Tetrasphaera, Sphingopyxis, Dechloromonas, Flavobacterium, Ohtaekwangia* (Świątczak and Cydzik-Kwiatkowska, 2018), *Meganema, Thauera, Paracoccus, Zoogloea* (Szabó et al., 2017), *Denitratisoma, Novosphingobium, Planctomyces, Ferruginibacter, Hyphomicrobium, Steroidobacter* (Yuan et al., 2019). Furthermore, Ali et al. reported that the bacterial compositions among different-sized aggregates are significantly different, and this dissimilarity increases with the increase in aggregate sizes, indicating an important role of species sorting in AGS systems (Ali et al., 2019). One exciting aspect of the application of AGS is the removal of pharmaceutical and personal care products in wastewater (Xia et al., 2014; Zhao et al., 2014; Wang et al., 2016). For instance, *Chryseobacterium, Actinotignum, Lactococcus, Shinella, and Clavibacter* have been facilitating the removal of tetracycline (Wang et al., 2018). The removal of prednisolone, naproxen, sulphamethoxazole and ibuprofen was enabled by antibiotic-resistant genes carrier such as *Firmicutes* sp., *Aeromonas* sp. and *Nitrospira* sp. (Xia et al., 2014). As such, future optimisation of AGS technology relies on the better understanding of the physiology, function, and interaction of the species for more efficient separation, maintenance, and treatments.

Although AGS has been widely applied in wastewater treatment to replace the traditional AS, it is not an energy cost-effective process (Foladori et al., 2018). Microalgal-bacterial aggregates have been extensively investigated and regarded as a greener technology during the last two decades. The photosynthetically produced oxygen by microalgae support the growth of bacteria, which in return produce CO_2_ for microalgal photosynthesis. Together, the whole consortium substantially remove the organic matter and nutrients in wastewater, with excellent settling characteristics allowing for biomass harvesting by gravity sedimentation (Quijano et al., 2017). While the genera such as *Chlorella* and *Scenedesmus* have been reported to be a suitable algal species (Gutzeit et al., 2005; Lee et al., 2013; Mezhoud et al., 2014; Vandamme et al., 2014; Shen et al., 2015; Kinnunen and Rintala, 2016; Arcila and Buitrón, 2017), so far, most bacteria identified in wastewater treatment using microalgal-bacterial systems belong to the phyla *Bacteroidetes* and *Proteobacteria*, such as *Betaproteobacteria, Bacteroidia, Flavobacteria, Gammaproteobacteria* (Su et al., 2011), *Sphingobacteria, Terrimonas*, and *Hyphomon-as* (Lee et al., 2013). More detailed knowledge on the interaction between different algal-bacteria species would facilitate better design.

## *Tiny Size, Big Trouble*—The *in vivo* Bacterial Aggregates

### Cystic Fibrosis (CF) Airway Infection

**FIGURE 3:**
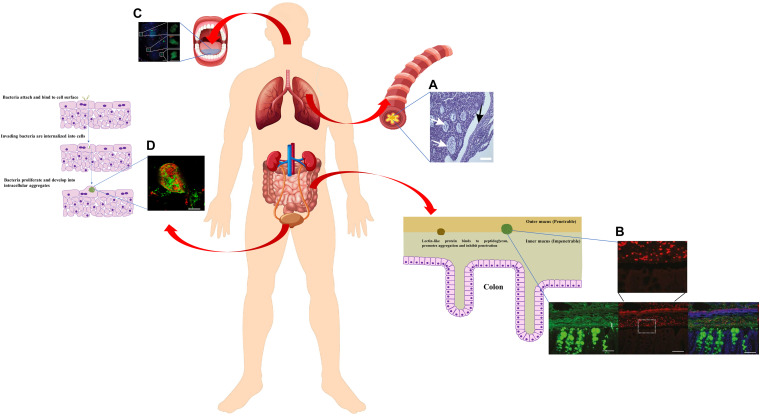
Schematic diagram of bacterial aggregates found in human body (constituted using vectors available from Vecteezy.com). Zoomed-in schematic of colon cells and mucus layer with bacterial aggregates was adapted from Donaldson et al. (2015). Zoomed-in schematic of bacterial invasion and proliferation in bladder urothelium cells was adapted from Rosen et al. (2007) (Donaldson et al., 2015). **(A)** A thin section of a freshly excised, obstructed CF bronchus stained with hematoxalin/eosin. *P. aeruginosa* macrocolonies are localised in intraluminal material (white arrows), but absent from epithelial surface (black arrow) (Worlitzsch et al., 2002). **(B)** Carnoy-fixed normal distal colon immunohistochemistry of the mucus layer (anti-MUC2, green) and Gram-positive bacteria (anti-LTA antibody, red) (Bergström et al., 2016). **(C)** Epifluorescence picture of planktonic bacterial aggregates in fresh human saliva (blue, cell surface of bacteria and eukaryotic cells; red, dead cells; green, general DNA staining). Roughly spherical aggregates were shown as close-up areas (Itzek et al., 2017). Scale bar 20 μm. **(D)** CLSM micrograph of intracellular bacterial aggregates in urine samples from women with cystitis, stained with antibodies against *E. coli* (green) and uroplakin III (red) (Rosen et al., 2007). Written permissions to re-use images were obtained from respective authors, journals, and/or Copyright Clearance Centre when required.

As the major contributor to morbidity and mortality in cystic fibrosis patients with chronic airway infection, *P. aeruginosa* has been extensively studied for its physiology *in vivo*. While it is well-established that *P. aeruginosa* forms substantial biofilms *in vitro*, whether or not they adhere to epithelial cells in CF patients has been controversial. Some earlier studies using cultured epithelial cells tended to support the adherence theory. Imundo et al. (1995) reported that tetrasaccharide of αGM is a receptor for *P. aeruginosa* and its increased abundance in the apical membrane of CF epithelia most likely contributes to *P. aeruginosa* colonisation in the CF lung. Hahn (1997) further reported that it was the type IV pili of *P. aeruginosa* that was responsible for binding to αGM. Interestingly, *P. aeruginosa* was shown to not avidly bind to normal, uninjured epithelial cell surfaces, but the binding of *P. aeruginosa* to inflamed or injured epithelial cells (CF or mechanically ventilated patients) is significantly higher than to normal cells (De Bentzmann et al., 1996). However, in recent years, another theory emerged from CF *ex vivo* studies that has become more and more popular, supporting the fact that bacteria do not necessarily attach to surfaces for the establishment of biofilm-like aggregates that contribute to chronic infections (Campodónico et al., 2008; Alhede et al., 2011; Hall-Stoodley et al., 2012). For instance, *P. aeruginosa* can enter airway epithelial cells and internalised bacteria form clusters within airway cells, favouring their persistence that results in the difficulties in the treatment of airway infection (Garcia-Medina et al., 2005). A number of studies claimed that *P. aeruginosa* was never seen localised to the epithelial cell surface in CF airways when sputum and explanted/thin sections of airways from CF patients were examined. Instead, bacterial aggregates were found bound to mucus and surrounded by polymorphonuclear neutrophils (Figure 3A; Hassett et al., 2002; Worlitzsch et al., 2002; Bjarnsholt et al., 2009). Using artificial sputum medium, Sriramulu et al. (2005) discovered that *P. aeruginosa* form tight microcolonies, or aggregates attached to sputum components, which can be enhanced by the amino acid content via quorum sensing pathways (Sriramulu, 2010; Williams and Davies, 2012). By using mucus gels containing *ex vivo* sputum from patients, Staudinger et al. (2014) revealed it is neutrophil elastase in CF sputum that promotes the formation of bacterial aggregates. This was further evidenced by Caceres et al. (2014) showing that the incorporation of human neutrophil-derived products enhanced the formation of non-attached *P. aeruginosa* aggregates. The self-aggregating, non-attached *P. aeruginosa* grown in artificial CF sputum resemble those observed in the CF lung sputa both transcriptionally and phenotypically, suggesting the aggregates embedded in mucus are likely to be the prevalent growth mode at chronic infection sites (Fung et al., 2010). It is worth noting that although non-attached *P. aeruginosa* aggregates were proved to resemble surface attached biofilms in growth rate, internal structures of the matrix, tolerance to antibiotics and resistance to phagocytes (Alhede et al., 2011; Caceres et al., 2014), mutants that inhibit biofilm formation on surfaces still allow for aggregation in mucus under the force of abundant polymer (Staudinger et al., 2014; Secor et al., 2018). Furthermore, isolates taken from chronic CF infections are often defective in biofilm formation in standard plate assays, suggesting that the strains capable of forming aggregates *in situ* may not attach well to surfaces (Lee et al., 2005; Deligianni et al., 2010). The production mechanisms of non-attached aggregates embedded in polymers may be very different from surface-attached biofilms, depending on the passive “depletion aggregation” driven by entropic forces generated by polymers rather than the bacterial activities (Secor et al., 2018). Yet, such observations opened the door for fundamental research on the formation and dispersal of suspended bacterial aggregates in polymer rich environments resembling the chronic infection sites *in vivo*, which are more clinically relevant than microtiter plates or 2D epithelial cells for the development of better treatment strategies.

### Chronic Wound Infection

Growing evidence shows that bacterial aggregates found in chronic wounds tend to be entrapped in wound beds or compromised soft tissues with extensive host polymers and debris (James et al., 2008; Pabst et al., 2016). Relatedly, different bacterial strains such as *Pseudomonas aeruginosa, Staphylococcus epidermidis* and *Probionibacterium acnes*, were found to form aggregates in soft tissue dermal fillers, exhibiting tolerance to antibiotics once established (Alhede et al., 2014). This implies that the highly hydrated *in vivo* environment filled with extracellular matrix such as collagen and elastin may be favoured by bacterial aggregation. When looking into biopsied leg ulcer samples from different patients, *S. aureus* are more frequently present on or near the wound surfaces, whereas *P. aeruginosa* are largely deep buried inside the wound beds surrounded by polymorphonuclear neutrophils (Kirketerp-Møller et al., 2008; Fazli et al., 2009). Moreover, *P. aeruginosa* aggregates were found to be more prevalent in diabetic wounds compared to non-diabetic wounds. They were exclusively found within the wound bed and at the wound margin, leading increased tolerance to antibiotics and impaired healing (Watters et al., 2013). Morgan et al. (2019) showed that human wound infection isolates do not form substantial biofilms *in vitro*. The authors further demonstrated that genetically modified *P. aeruginosa* strains defective in biofilm formation can still form aggregates in the wounds using a murine model (Morgan et al., 2019). This may indicate that bacterial aggregates imbedded in secretions, not solid surface-attached biofilm, may be a default growth mode driven by thick polymer-rich surrounding as a consequence of host conditions. Specific mechanisms required for the formation of aggregation may be needed *in vivo*, which do not overlap with biofilm formation functions required *in vitro* (Morgan et al., 2019).

### Other Body Fluids

Mucus is also found in intestines. *Salmonella typhimurium* was shown to aggregate and bind to colonic mucus, which is mediated by a 66-Kd heat shock protein produced by the bacteria themselves (Ensgraber and Loos, 1992). At the same time, the host can produce abundant lectin-like protein ZG16 (zymogen granulae protein 16) in distal colonic mucus, which binds to the bacterial cell wall peptidoglycan and induces the aggregation of Gram-positive bacteria (Figure 3B; Bergström et al., 2016). As such, both bacteria and host develop mechanisms to trigger bacterial aggregation for keeping bacteria a safe distance from the colon epithelium and preventing inflammation (Bergström et al., 2016).

Interestingly, while the first description of the biofilm concept came from Leeuwenhoek examining his own scraped-off dental plaque (Høiby, 2017), saliva has been reported to facilitate the aggregation of bacteria from a number of different genera, including species such as *Actinomyces israelii, Actinomyces naeslundii, Actinomyces viscosus, Bacteroides intermedius, Bacteroides gingivalis, Streptococcus cricetus, Streptococcus mutans, Streptococcus rattus, Streptococcus sanguis, Streptococcus sobrinu*s, *Streptococcus intermedius, Streptococcus gordoni* (Rosan et al., 1982; Golub et al., 1985; Koop et al., 1989; Yamaguchi, 2004; Kitada and Oho, 2012; Itzek et al., 2017). It was suggested that the interactions between salivary IgA and bacterial surface components cause bacterial aggregation, which also depends on various factors (Koop et al., 1989; Yamaguchi, 2004). As a result, saliva blocks the adherence of bacteria onto surfaces and promotes the clearance from the oral cavity. More recent studies further revealed that bacterial cells are aggregated into an ideal size by saliva for the recognition by polymorphonuclear neutrophil granulocytes, which then de-aggregate them through serine proteases for more efficient phagocytosis and killing (Figure 3C; Itzek et al., 2017). Using this elaborate pathway of modulating bacterial aggregation, saliva plays a significant role in first line defence preventing infections and damage in the bloodstream and tissues.

Uropathogenic bacterial aggregates, also known as intracellular bacterial communities, were found in planktonic form in urine from both healthy volunteers and urinary tract infection patients (Reid et al., 1990; Rosen et al., 2007; Robino et al., 2014; Conover et al., 2016). It was suggested that *E. coli* invades the superficial bladder cells, which are then exfoliated and removed with the flow of urine as a response to the invasion (Mulvey et al., 2001). However, bacteria find their way to multiply intracellularly and eventually establish biofilm-like aggregates (Figure 3D; Rosen et al., 2007; Robino et al., 2014; Conover et al., 2016). Also similar to biofilms growth stages, these aggregates progress through four stages, with distinct growth rates, bacterial lengths, colony organisations, motility, and dispersal (Justice et al., 2004). With evolutionary development through different stages, some physiologically adapted, filamentous intracellular bacteria could emerge from the aggregates (Mulvey et al., 2001; Justice et al., 2004). As such, these aggregates generate a dormant reservoir of pathogens inside the bladder cells, allowing for the escape from host clearance and are tolerant to antibiotics (Anderson et al., 2004). Once dispersed, the re-emerging cells may cause the recurrence of urinary tract infection, and can also be associated with chronic cystitis (Anderson et al., 2004; Schwartz et al., 2011; Robino et al., 2014). Once again it is aggregated bacteria rather than biofilms attached to solid surfaces that are causing these intractable medical problems.

## *Modern Times*—Novel Technologies in Aggregate Research

### *In vitro* Models

**FIGURE 4:**
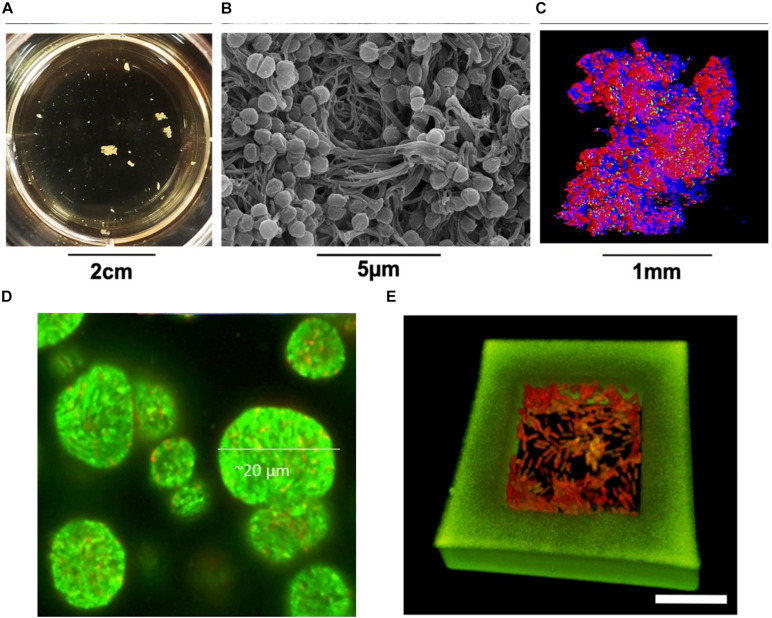
*S. aureus* aggregates cultured in human synovial fluid (37°C incubation, microaerophilic chamber, 120 rpm) and photographed **(A)** in the 24-well plates, **(B)** by SEM, and **(C)** by CLSM (Gilbertie et al., 2019). **(D)** CLSM micrograph of GFP-tagged *P. aeruginosa* PAO1 aggregates (green) grown in alginate beads (Sønderholm et al., 2017). **(E)** *P. aeruginos*a cells contained in a 3D-printed microtrap. Scale bar = 10 μm. Green, the walls of microtrap; Red, the bacteria (Connell et al., 2014). Written permissions to re-use images were obtained from respective authors, journals, and/or Copyright Clearance Centre when required.

Each model has distinct limitations reflecting the true situation under natural or clinical settings, including many *in vivo* models. *In vitro* studies are still largely applied for their easier approach and modulation. Some modifications on traditional biofilm research protocols have proved to stimulate non-attached bacterial aggregation, which mimic the structure and characteristics of *in vivo* biofilm infection better than the specific surface attachment using defined conditions. Even just altering liquid medium can yield some surprising results. For example, Schleheck et al. (2009) found *P. aeruginosa* aggregates in shaking planktonic batch cultures using just mineral salts medium containing a limited carbon-resource. Equine or porcine synovial fluid was shown to be a successful model for growing suspended *S. aureus* aggregates to mimic human joint infections (Figures 4A–C; Gilbertie et al., 2019). Bolton broth containing 50% plasma and 5% freeze-thaw laked horse red blood cells (defibrinated) was used to study polymicrobial biofilms/aggregates in wound infection (Dalton et al., 2011). Synthetic sputum medium was shown to promote the formation of *P. aeruginosa* aggregates with sizes comparable to those observed in CF lungs (Darch et al., 2017). Agarose gel seeded with *S. aureus* and alginate beads inoculated with *P. aeruginosa* were developed to mimic the aggregates formed in the lung mucus or in dermal wounds matrices (Figure 4D; Pabst et al., 2016; Sønderholm et al., 2017). Apart from the relatively rough immobilisation methods to achieve bacterial aggregates as mentioned above, a much more refined method was developed using 3-D printed bacterial microtraps to control the spatial distances between bacterial aggregates (Figure 4E; Connell et al., 2014). The application of this elegant technique with proper biomarkers allows for both the control of cell number in the aggregates and the real-time monitoring of quorum sensing communication between communities, which significantly enhances the accuracy of aggregate-related studies (Connell et al., 2014). Artificial wound models have also been developed, containing a dermis-like scaffold composed of hyaluronic acid and collagen immersed into wound-like medium consisting of Bolton broth supplemented with 50% heparinised bovine plasma and 5% freeze-thaw laked horse blood (Grassi et al., 2019). These recent *in vitro* models have been proved to be more clinically relevant, hence are ideal for bacterial aggregation studies.

### Quantitative Software and Methods

Both descriptive and quantitative methods have been documented to describe bacterial aggregates under different settings. Microscopic or macroscopic images can be directly shown for the visual comparisons (Baugh et al., 2014; Foulston et al., 2014; Wessel et al., 2014; Jagmann et al., 2015; Zeng et al., 2015; Guo et al., 2016). Optical density (OD) values have been used for indirect measurements. For example, Guo et al. (2016) used low speed centrifugation to separate cell aggregates and planktonic cells. Cell aggregates were then collected for vortexing and CFU counting so that the cell number in aggregates was enumerated (Guo et al., 2016). Baugh et al. (2014) grew bacterial aggregates in a static system and compared the OD value of the suspension below the aggregating surfaces. With the same initial inoculum and growth rate, the lower the OD value of suspension (planktonic cells), the higher the aggregation levels (Baugh et al., 2014). Naturally, OD values do not directly reflect the characters of the cell aggregates themselves. Several quantification methods have been developed to calculate 2D micrographs. For example, software such as ImageJ^TM^ can be applied to change the aggregative structures in each image to a binary code and then the pixel number can be measured to represent the structure size (Jung et al., 2015). Stoodley et al. (2001) used image subtraction to change the grey scale of background so that the areas and diameters of cell aggregates can be measured, from which the means and standard deviations of the aggregate sizes can be calculated. With more advanced equipment, Schleheck et al. (2009) used laser diffraction analysis to determine the exact diameters of cell aggregates and different size ranges were grouped into different catagories. Similarly, a coulter counter measuring electric current change was applied to reflect the size of the particles (Zhang et al., 2013) and a cell counter can be used to count individual cells within cell aggregates (Dastghey et al., 2015). While data acquired from these devices are mostly numerical data, CLSM is a popular approach for 3-D microscopic outputs. Accordingly, different softwares have emerged to analyse 3-D bacterial structures. COMSTAT was designed for biofilm research in 2000 (Heydorn et al., 2000). Although it does calculate the volumes of microcolonies/aggregates, the microcolonies are defined as connected to the substratum (Heydorn et al., 2000) and hence, for non-attached aggregates in liquid cultures, this classic software may not be ideal. Another software, *daime*^TM^ (digital image analysis in microbial ecology), was designed several years later mainly tackling 2-D and 3-D micrographs acquired from environmental and medical samples using FISH probes. 2-D and 3-D objects in images (stacks) can be automatically identified by the software package *daime*, which are then used to quantify microbial populations and even evaluate new FISH probes. The quantification of spatial localisation patterns of microorganisms in complex structures like biofilms is also available (Daims et al., 2006). Over the years, ImageJ^TM^ has become more and more powerful with additionally coded plugins. The 3D Objects Counter and 3D Manager function from the 3-D ImageJ Suite plugin (Ollion et al., 2013) are suitable for identifying bacterial aggregates in CLSM micrographs. Similarly, another powerful software named Imaris can be used to automatically identify and quantify different parameters of 3-D aggregates, such as surface areas, diameters, and volumes. It also offers functions such as particle tracking and segmentation, which are very useful if the researcher is only interested in a particular aggregate. While Imaris is less accessible due to its expensive package and maintenance price, an open source software, BiofilmQ, also allows for processing micrographs acquired from different fluorescent microscopies (Hartmann et al., 2019). Different parameters, such as size, volume, location, distance, fluorescent intensity, and even correlation of co-localisation, can be measured in any spatially structured microbial community including microscopic, mesoscopic, macroscopic colonies, biofilms, and non-attached aggregates (Hartmann et al., 2019). Recently, a novel index, Aggregation Coefficient, was developed to more directly quantify the bacterial aggregation levels using CLSM micrographs, MATLAB and ImageJ macro (Cai et al., 2019). Each CLSM stack of images can generate a single numerical index, which makes comparison between large scale samples easier. As many breakthroughs in biological science rely on novel inventions and conquering technical bottlenecks, improvements on both microscopes imaging and the accompanying computational programs will significantly enhance our perspectives on microbial worlds.

## Conclusion and Final Thoughts

Bacterial aggregates are ubiquitous, but they have been considered as an alternative format of surface-attached biofilms and thus underestimated for years. In this review, the existence of non-surface attached bacterial aggregates discovered from ocean, fresh water, wastewater treatment systems, as well as chronic infection sites were collectively presented, providing new perspectives and questions based on some fundamental similarities or differences. For instance, bacterial aggregates formed in MGP were reported to rely on the sticky nature of polymers produced by planktons (Rochelle-Newall et al., 2010); host secreted abundant polymers found in chronic infection sites may drive bacterial aggregation through entropic forces independent of biofilm formation (Secor et al., 2018); lectin-like protein in colon mucus binds to cell wall peptidoglycan and thus aggregates the bacteria that might otherwise cause inflammation if not kept at a safe distance from epithelium (Bergström et al., 2016). It seems that physical, chemical, and biological mechanisms may all be involved in polymer-induced bacteria aggregation. Are they all contributing to bacterial aggregation in different settings as generic mechanisms, or do different polymers act distinctly due to their specific nature? Are different bacterial proteins/polysaccharides playing important roles in how the aggregates are formed? While extensive investigations have been made towards how solid surfaces can change biofilm initiation and formation, future work may focus on the mechanisms of how different polymers influence bacterial aggregation. More efficient intervention strategies in chronic infection may then be applied to disperse or separate aggregates developed in mucus. Additionally, it may also help us to better design polymer used in medical settings such as dermal fillers, which have been shown to support bacterial aggregation (Alhede et al., 2014).

The overuse of antibiotics in human and animal husbandry is now widely acknowledged to generate antibiotic resistant strains and consequently lead to treatment failure. The formation of biofilms and aggregates adds another layer of complexity, both causing antibiotic tolerance (Olsen, 2015) and promoting horizontal resistant gene transfer (Madsen et al., 2012; Savage et al., 2013). While minimal inhibition and bactericidal concentrations are easy to identify in laboratories for either planktonic cells or biofilms from pure cultures, real-life situations are much more complicated and thus bring in another issue—treatment concentrations. Numerous reports showed that sublethal (sub-MIC) concentrations of antibiotics can actually promote biofilm formation *in vitro* (Kaplan, 2011; Andersson and Hughes, 2014), although the detailed mechanisms may vary and are yet to be clarified for different strains and compounds. A similar phenomenon is also found in freshwater bacterial isolates, where antibiotics can promote bacterial aggregation (Corno et al., 2014). Will the exposure to sub-MIC antibiotics lead to the growth of bacterial aggregates *in vivo*? Is the MIC for surface-attached biofilms the same for non-attached aggregates formed by the same strains? More direct evidence is required to confirm these speculation, and distinguishing aggregates from biofilms will more accurately guide the proper usage of antibiotics in clinical settings.

Are antibiotic resistance and biofilm tolerance totally negative? Antibiotics are documented to occur in aquatic environmental settings such as wastewater treatment plants, rivers, and lakes as a result of health care and animal husbandry, and this pollution can in turn accelerate the emergence of new antibiotic-resistant pathogens dangerous to human and animal health (Gothwal and Shashidhar, 2015). However, antibiotic-resistant strains were found to be retained in bacterial aggregates in the membrane biological reactors, serving a key function in facilitating the removal of antibiotic pharmaceuticals and personal care products in the wastewater (Xia et al., 2014). A list of antibiotic destructases have been found from environmental strains to inactivate different categories of antibiotics, of which some have also been found in human pathogens (Crofts et al., 2017; Markley and Wencewicz, 2018). Is it possible that we can use sludge intentionally seeded with antibiotic resistant strains or engineered enzymes to eliminate antibiotic pollutants in drinking water in the future? Will the mechanistic studies of antibiotic degradation lead to the development of novel adjunctive therapeutic agents that can suppress the antibiotic-resistance in clinically relevant pathogens, such as anhydrotetracycline that inhibits tetracycline destructase (Park et al., 2017)? Will these novel agents influence the development of aggregates at the same time with lowering antibiotic resistance? Much more awaits to be revealed, but the rich environmental bacteria reservoir never fails to surprise us—from the discovery of novel antibiotics, to the degradation of unwanted antibiotics.

In summary, non-surface attached bacterial aggregate are a secret masked ball full of excitement and opportunities to be explored. Thanks to the extensive studies in attached biofilms, lots of mechanistic pathways, treatments, and tools may also be applied for non-attached aggregates with some modifications. Nature has always inspired us for novel inventions and discoveries, which can also be the case in our understanding and co-inhabitation with the bacterial kingdom. Hence, more substantial research in environmental particles and flocs may inspire and facilitate clinical studies, now that we can demonstrate that aggregation not only happens in aquatic environmental settings, but also in CF mucus, wound infection sites and in other body fluids. Although much remains to be known, it is the aim of this review to bridge the gaps in knowledge that exist among researchers in different fields and shed light on some potential research directions for future work.

## Author Contributions

Y-MC did the bibliographic review and wrote the manuscript.

## Conflict of Interest

The author declares that the research was conducted in the absence of any commercial or financial relationships that could be construed as a potential conflict of interest.
